# Relationship between epithelial cell adhesion molecule (EpCAM) overexpression and gastric cancer patients: A systematic review and meta-analysis

**DOI:** 10.1371/journal.pone.0175357

**Published:** 2017-04-12

**Authors:** Meng Dai, Fei Yuan, Cuiqun Fu, Guodong Shen, Shilian Hu, Gan Shen

**Affiliations:** 1 Department of Geriatrics, Anhui Provincial Hospital affiliated to Anhui Medical University, Hefei, Anhui, China; 2 Anhui Provincial Key Laboratory of Tumour Immunotherapy and Nutrition Therapy, Hefei, Anhui, China; 3 Department of Pharmacy, Yijishan Hospital of Wannan Medical College, Wuhu, Anhui, China; The Ohio State University, UNITED STATES

## Abstract

**Objectives:**

The epithelial cell adhesion molecule (EpCAM) is one of the most commonly used markers of cancer stem cells (CSCs), but the clinical and prognostic significance of EpCAM in gastric cancer (GC) remains disputable. Motivated by heterogeneous and inconclusive results, we conducted a systematic review and meta-analysis to systematically summarize and elucidate the association between EpCAM overexpression and GC patients.

**Methods:**

The PubMed, Cochrane Library, Medline, Web of Knowledge and the China National Knowledge Infrastructure (CNKI) databases were searched to identify relevant studies. The RevMan 5.3 software was used for the meta-analysis. Fixed-effects or random-effects models were applied depending on the presence of heterogeneity. The pooled odds ratio (ORs) and 95% confidence intervals (CIs) were applied to estimate the associations between EpCAM and gastric cancer. For the significant heterogeneity studies, sensitivity analyses were applied based on the population to test the robustness of the pooled results and identify possible sources of heterogeneity.

**Results:**

A total of 11 studies including 1960 GC patients met our inclusion criteria. The results of the meta-analyses revealed that there were significant differences in EpCAM overexpression and tumour size (OR = 2.97, 95% CI: 2.13~4.13, P < 0.00001), the nature of the tissue (OR = 80.30, 95% CI: 29.21~220.81, P < 0.00001), lymph node metastasis (OR = 2.78, 95% CI: 1.23~6.27, P = 0.01), and the cumulative 5-year overall survival rate (OR = 0.54, 95% CI:0.29~0.99, P = 0.05). No significant associations were identified between EpCAM overexpression and gender (OR = 0.89, 95% CI: 0.66~1.19, *P* = 0.43), age (OR = 1.13, 95% CI: 0.58~2.20, *P* = 0.73), tumour stage (OR = 2.26, 95% CI: 0.79~6.45, P = 0.13), distant metastasis (OR = 2.15, 95% CI: 0.20~22.69, P = 0.52), TNM stage (OR = 5.14, 95% CI: 0.77~34.37, *P* = 0.09), Lauren type (OR = 1.18, 95% CI: 0.08~16.45, P = 0.9), differentiation (OR = 1.88, 95% CI: 0.65~5.41, P = 0.24). However, due to significant heterogeneity in tumor stage, lymph node metastasis, TNM stage, differentiation and Lauren type, these results should be taken carefully.

**Conclusions:**

The meta-analysis demonstrated that the expression of EpCAM in the gastric cancer group was greater than that in the control group. Moreover, EpCAM overexpression was associated with larger tumour size, lymphnode metastasis and worse prognosis in gastric cancer. Due to significant heterogeneity, the sensitivity analysis suggests that population factor may be an important source of heterogeneity, and these results should be treated with caution. EpCAM may be useful as a novel prognostic factor, and large-scale and well-designed studies are needed to validate our results in the future.

## Introduction

According to a global statistical report from 2015[1], an estimated 951,600 new gastric cancer (GC) cases and 723,100 deaths occurred in 2012. GC ranks fourth in terms of incidence and second in mortality among all cancers worldwide. Generally speaking, the incidence ratesare highest in Eastern Asia (particularly in China, Korea, Japan, and Mongolia) and lowest in Northern America and the majority of Africa. Conventional treatments for GC include surgery, radiotherapy and chemotherapy, which play important roles in the early stages of GC; however, the recurrence rate following curative surgery has been reported to be 40–60%[2]. Although diagnosis and treatment greatly improves the 5-year survival rate of GC patients, this rate is still less than 20% and is particularly bad for more advanced stages[3–4].

In early 21st century, CSCs became a hot spot in the field of cancer research[5]. Gastric cancer stem cells (GCSCs) are a small group of gastric cancer cells that possess self-renewal, proliferation and differentiation potentials[6]. These cells cannot be killed by current chemotherapy, radiotherapy or other anti-cancer treatments. A target that is currently being explored in GC is the epithelial cell adhesion molecule (EpCAM or CD326). EpCAM is a 40-kDa type I transmembrane glycoprotein that is known to be highly expressed in epithelial carcinomas and serves as a prognostic factor[7]. Several studies pointed that EpCAM was identified as a one of tumour stem cell markers and an potential target for cancer therapy[8]. However, EpCAM was not considered as a tumour stem cell marker because it appeared to be associated with a more favorable prognosis in other studies[9]. As the correlations of the overexpression of the EpCAM with gender, age, various clinicopathological features, and the overall survival rate of GC patients have not been systematically reported. Here, based on the current researches, we performed a systematic review of the literatures and a meta-analysis to determine the above-mentioned associations.

## Materials and methods

### Literature search strategy

Related articles were identified by searching the PubMed, Cochrane Library, Medline, Web of Knowledge and CNKI databases. The last search was updated April 10, 2016. The search strategy included the following terms: EpCAM, CD326, stomach, gastric, neoplasm, cancer, etc. The reference lists of the relevant articles were also screened to further identify potential studies.

### Inclusion and exclusion criteria

Two authors (Dai and Yuan) searched and selected theeligible studies. Studies were included if they meet the following criteria: (1) evaluations of the association between EpCAM and gastric cancer, (2) case-control and cohort studies, (3) studies focusing on human beings, (4) diagnoses of GC proven by immunohistochemical (IHC) methods, and (5) detailed data were available to calculate the odds ratios (ORs) and 95% CIs. The exclusion criteria were the following: (1) duplications of previous publications; (2) case reports, letters, reviews, meta-analysis, editorial articles and animal trials; (3) studies with incomplete or non-detailed or non-usable data. When multiple publications on the same study population were identified, only the largest or most informative study was included in this meta-analysis.

### Data extraction and quality assessment

Data tables were made to extract all of the relevant data from texts, tables and figures of each of the included studies. Two observers (Dai and Yuan) independently extracted the following information: the first author, year of publication, patient number, sex ratio, median age, positive cases of high EpCAM expression, Newcastle-Ottawa scale (NOS) scores and reference number. The quality of each study was assessed using the NOS. The NOS uses a “star” rating system to judge quality that is based on three aspects of the study, i.e., selection, comparability, and exposure or outcome [10].

### Statistical analysis

The association of EpCAM overexpression with gender, age, 5-year overall survival and other clinicopathological conditions of GC, such as tumour size (>5 cm vs. ≤5 cm), the nature of the tissue (gastric cancer vs. control tissue), tumour stage (T3~T4 vs. T1~T2), lymph node status (positive vs. negative), distant metastasis (positive vs. negative), TNM stage (III/IVvs.I/II), differentiation (poor vs. well/moderate), and Lauren type (intestinal group vs. diffuse group) were estimated by calculating ORs with the 95% CIs. The meta-analyses were performed using RevMan 5.3 software. Heterogeneitywas assessed with the Q statistic and the I^2^statistic. P < 0.05 and I^2^> 50% were considered indicative of statistically significant heterogeneity[11]. When significant heterogeneity was present, random-effects models were used for the meta-analysis; otherwise, fixed-effects models were used. We used the ORs and 95% CIs to evaluate the correlations of EpCAM overexpression with a series of parameters. For the significant heterogeneity studies, we conducted sensitivity analyses based on Chinese population and non-Chinese population to test the robustness of the pooled results and identify if population factor influenced meta-analytic estimates. We graphically assessed publication bias using funnel plots and quantitatively assessed bias using Begg's test and Egger's test. P < 0.05(two-sided) was considered indicative of statistically significant publication bias.

## Results

### Literature information

**Fig 1:**
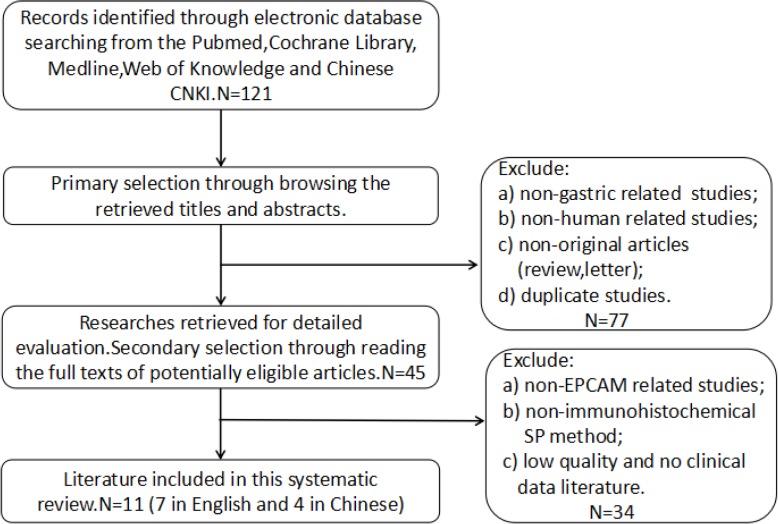
Flow chart for selection of the studies.

The process of the selection of the studies included in this meta-analysis is presented in Fig 1. A total of 121 studies were retrieved from the PubMed, Cochrane Library, Medline, Web of Knowledge and CNKI databases. Based on reading of the titles and abstracts, 77 of those studies were excluded because they were non-gastric-related studies, non-human-related studies, non-original articles (reviews and letters) or duplicate studies. Regarding the remaining studies, based on reading of the full texts, we excluded 34 studies because they were non-EpCAM-related studies, employed non-immunohistochemical SP methods, were of low quality or provided no clinical data. Ultimately, 11(7 in English and 4 in Chinese) eligible studies [12–22] that met the inclusion criteria were included in our meta-analysis.

### Study characteristics

The main characteristics of the 11 included studies are presented in Table 1. Among these studies, 1 of 428 GC patients originated from the Switzerland, one of 163 originated from Germany, 1 of 99 originated from Korea, 2 of 310 originated from the Netherlands, and 6 of 960 originated from China. A total of 1960 GC patients were included in this meta-analysis, including 1381 who were EpCAM-positive and 579 who were EpCAM-negative based on IHC. All detected specimens were derived from gastric cancer tissues acquired via surgical resection or biopsy. The majority of the patients were males (58.2% from 9 studies). The median ages ranged from 52.5 to 70 years old (from 5 studies). The NOS results revealed that the score ranged from 7 to 9.

**Table 1:** Characteristics of the included studies.

Studies	Year	Country	Cases(n)	Sex (M/F)	Age	EPCAM expression rate (%)	NOS	reference number
Danielle	2001	Netherlands	30	NS	NS	28(93.3%)	7	12
Du	2009	China	100	61/39	NS	74(74%)	9	13
Feride	2013	Germany	163	106/57	70(34–91)	126(77%)	8	14
I Songun	2005	Netherlands	280	NS	64.7(31–84)	260(92.9%)	9	15
Lu	2011	China	91	70/21	NS	84(92.3%)	8	16
MEE JOO	2005	Korea	99	69/30	60	34(34.3%)	9	17
Peng	2011	China	31	18/13	NS	21(67.7%)	7	18
P Went	2006	Switzerland	428	117/311	59(27–92)	417(97.5%)	7	19
Wang	2013	China	601	428/173	NS	247(41.1%)	8	20
Yang	2012	China	95	66/29	NS	56(58.95%)	7	21
Zhang	2011	China	42	24/18	52.5(30–77)	34(81%)	8	22

Note: EpCAM, epithelial cell adhesion molecule; IHC, immunohistochemistry; NS, not specified.

### Results of the meta-analysis

#### 1. Gender of the GC patients: Male group vs. female group

**Fig 2:**
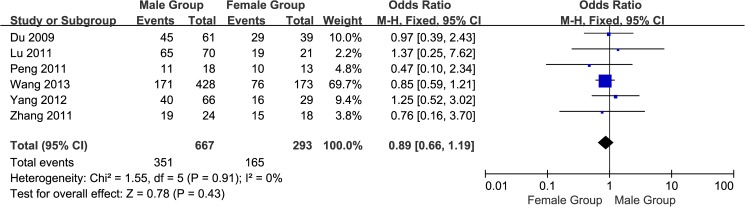
Meta-analysis of overexpression of EpCAM in male group and female group.

A total of six studies[13,16,18,20–22] reported EpCAM overexpression in the male and female groups. The I^2^ estimate indicated no heterogeneity (I^2^ = 0%) among the studies, and the fixed effects model used in this meta-analysis indicated no significant association of the overexpression of EpCAM with gender (OR = 0.89, 95% CI: 0.66~1.19, *P* = 0.43; Fig 2).

#### 2. Age of the GC patients:>60 group vs. ≤60 group

**Fig 3:**
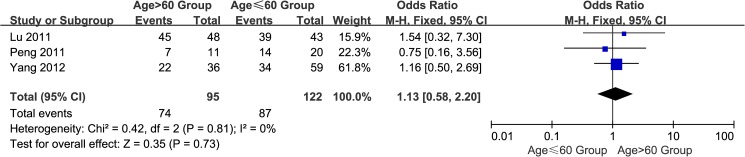
Meta-analysis of overexpression of EpCAM in age>60 group and age ≤60 group.

A total of three studies[16,18,21] reported EpCAM overexpression group above and below the age of 60. The I^2^ estimate revealed no heterogeneity (I^2^ = 0%) among the studies, and the fixed effects model used in this meta-analysis indicated no significant association between the overexpression of EpCAM and age (OR = 1.13, 95% CI: 0.58~2.20, *P* = 0.73; Fig 3).

#### 3. Tumour size of GC tissues: Tumours >5 cm group vs. tumours ≤5 cm group

**Fig 4:**
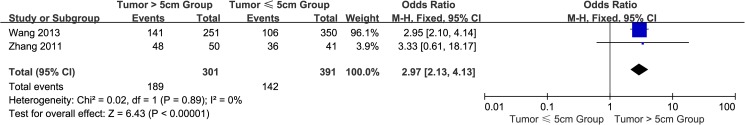
Meta-analysis of overexpression of EpCAM in tumour > 5cm group and tumour ≤5cm group.

A total of two studies[20,22] reported EpCAM overexpression in groups with tumour sizes >5 cm and ≤5 cm. The I^2^ estimate revealed no heterogeneity (I^2^ = 0%) among the studies, and the fixed effect model used in the meta-analysis indicated that expression of EpCAM in the tumour size >5 cm group was greater than that in the tumour size ≤ 5 cm group. The difference between the two groups was statistically significant (OR = 2.97, 95% CI: 2.13~4.13, P < 0.00001; Fig 4).

#### 4. Natures of the tissues: Gastric cancer group vs. control group

**Fig 5:**
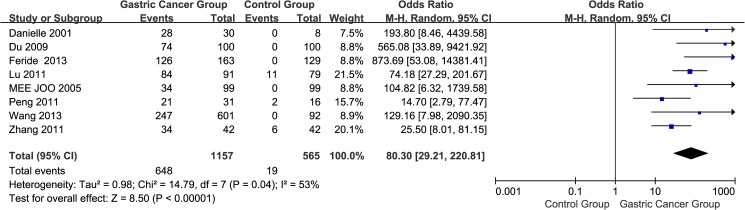
Meta-analysis of overexpression of EpCAM in gastric cancer group and control group.

A total of eight studies[12–14,16–18,20,22] reported EpCAM overexpression in gastric cancer groups (gastric cancer tissues) and control groups (pericarcinoma tissues or normal gastric tissues). The I^2^ estimate revealed significant heterogeneity (I^2^ = 53%) among the studies, and the random effects model used in thismeta-analysis indicated that the expression of EpCAM in the gastric cancer group was greater than that in the control group. The difference between the two groups was statistically significant (OR = 80.30, 95% CI: 29.21~220.81, *P* < 0.00001; Fig 5).

#### 5. Tumour stages of the GC tissues:T3~T4 group vs. T1~T2 group

**Fig 6:**
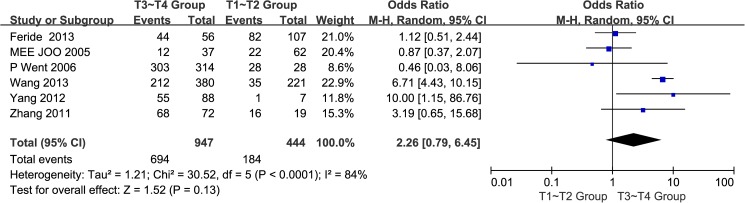
Meta-analysis of overexpression of EpCAM in T3~T4 group and T1~T2 group.

A total of six studies[14,17,19–22] reported EpCAM overexpressionin T3~T4 and T1~T2 groups. The I^2^ estimate indicated significant heterogeneity (I^2^ = 84%) among the studies, and the random effects model used in this meta-analysis indicated that there was no significant association between the overexpression of EpCAM and tumour stage(OR = 2.26, 95% CI: 0.79~6.45, *P* = 0.13; Fig 6). The results of sensitivity analyses showed that Chinese population were: I^2^ = 0%, OR = 6.49, 95% CI: 4.38~9.63, *P <*0.00001; non-Chinese population were: I^2^ = 0%, OR = 0.97, 95% CI: 0.55~1.71, *P =* 0.92 (S1 Fig).

#### 6. Lymph node (LN) metastases of the GC tissues: Positive group vs. negative group

**Fig 7:**
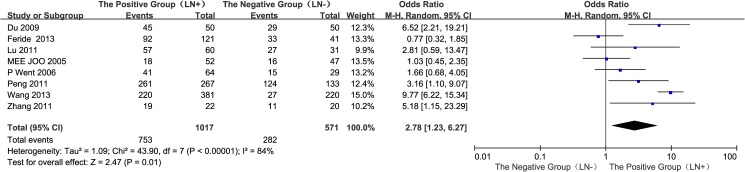
Meta-analysis of overexpression of EpCAM in LN (+) and LN (–) gastric cancer group.

A total of eight studies[13–14,16–20,22] reported EpCAM overexpression in positive and negative lymph node metastases groups of gastric cancer tissues.The I^2^ estimate indicated significant heterogeneity (I^2^ = 84%) amongthe studies, and the random effects model used in thismeta-analysisindicated that the expression of EpCAM in the positive group (LN+) was greater than that in the negative group (LN−). The difference between the two groups was statistically significant (OR = 2.78, 95% CI: 1.23~6.27, *P* = 0.01; Fig 7). The results of sensitivity analyses showed that Chinese population were: I^2^ = 30%, OR = 6.16, 95% CI: 3.63~10.44, *P <*0.00001; non-Chinese population were: I^2^ = 0%, OR = 1.09, 95% CI: 0.66~1.79, *P =* 0.74 (S2 Fig).

#### 7. Distant (D) metastasis of GC tissues: Positive group vs. negative group

**Fig 8:**
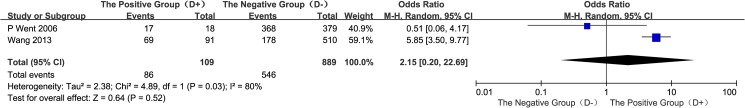
Meta-analysis of overexpression of EpCAM in D (+) and D (–) gastric cancer group.

A total of two studies[19–20] were included, one from Chinese population, the other from non-Chinese population, which reported EpCAM overexpression in D(+) and D(–) gastric cancer groups. The I^2^ estimate revealed significant heterogeneity (I^2^ = 80%) amongthe studies, and the random effects model used in thismeta-analysisindicated that there was no significant association between the overexpression of EpCAM anddistant metastasis (OR = 2.15, 95% CI: 0.20~22.69, *P* = 0.52; Fig 8).

#### 8. TNM stage of the GC tissues: III~IV stage group vs. I~II stage group

**Fig 9:**
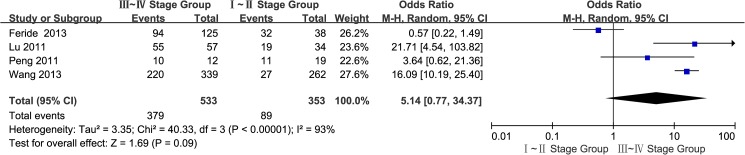
Meta-analysis of overexpression of EpCAM in Ⅲ~Ⅳ group andⅠ~Ⅱgroup.

A total of four studies[14,16,18,20] reported the EpCAM overexpression in III~IV and I~II groups. The I^2^ estimate revealed significant heterogeneity (I^2^ = 93%) among the studies, and the random effects model used in thismeta-analysisindicated that there was no significant association between the overexpression of EpCAM and the TNM stage(OR = 5.14, 95% CI: 0.77~34.37, *P* = 0.09; Fig 9). The results of sensitivity analysis showed that Chinese population were: I^2^ = 28%, OR = 13.69, 95%CI: 6.57~28.49, *P<*0.00001 (S3 Fig).

#### 9. Differentiation of GC tissues: Poorly differentiated group vs. well/moderate differentiated group

**Fig 10:**
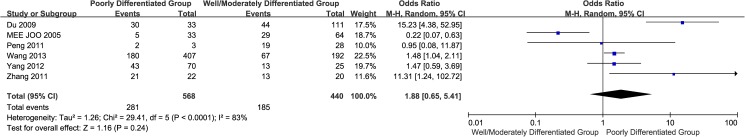
Meta-analysis of overexpression of EpCAM in poorly differentiated group and well/moderate differentiated group.

A total of six studies[13,17–18,20–22] reported the overexpression of EpCAM in poorly and well/moderate differentiated groups. The I^2^ estimate indicated significant heterogeneity (I^2^ = 83%) among the studies, and the random effects model used in thismeta-analysisindicated that there was no significant association between the overexpression of EpCAM anddifferentiation(OR = 1.88, 95% CI: 0.65~5.41, *P* = 0.24; Fig 10). The results of sensitivity analysis showed that Chinese population were: I^2^ = 11%, OR = 1.58, 95% CI: 1.02~2.44, *P =* 0.04 (S4 Fig).

#### 10. Lauren type GC tissues:Intestinal group vs. diffuse group

**Fig 11:**
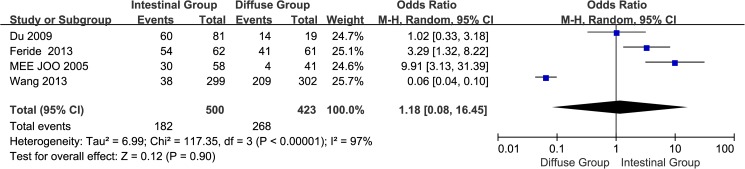
Meta-analysis of overexpression of EpCAM in intestinal group and diffuse group.

A total of four studies[13–14,17,20] reported EpCAM overexpression inintestinal and diffuse groups. The I^2^ estimate revealed significant heterogeneity (I^2^ = 97%) among the studies, and the random effects model used in thismeta-analysisindicated that there was no significant association between the overexpression of EpCAM and the Lauren type(OR = 1.88, 95% CI: 0.08~16.45, *P* = 0.90; Fig 11). The results of sensitivity analysis showed that non-Chinese population were: I^2^ = 54%, OR = 5.39, 95%CI: 1.84~15.84, *P =* 0.002 (S5 Fig).

#### 11. EpCAM overexpression and the 5-year overall survival rate

**Fig 12:**
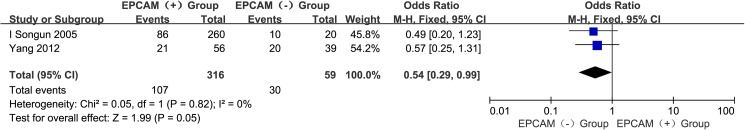
Meta-analysis of 5-year overall survival between EpCAM (+) and EpCAM (–) groups.

A total of two studies[15,21] reported the cumulative 5-year overall survival rates of the EpCAM-positive [EpCAM(+)] and EpCAM-negative [EpCAM(−)] gastric cancer patients. The I^2^ estimate revealed no heterogeneity (I^2^ = 0%) among the studies, and the fixed effects model used in thismeta-analysis indicated that the EpCAM-positive group suffered significantly worse prognoses than the EpCAM-negative group. The difference between the two groups was statistically significant (OR = 0.54, 95% CI:0.29~0.99, *P* = 0.05; Fig 12).

#### Publication bias

**Fig 13:**
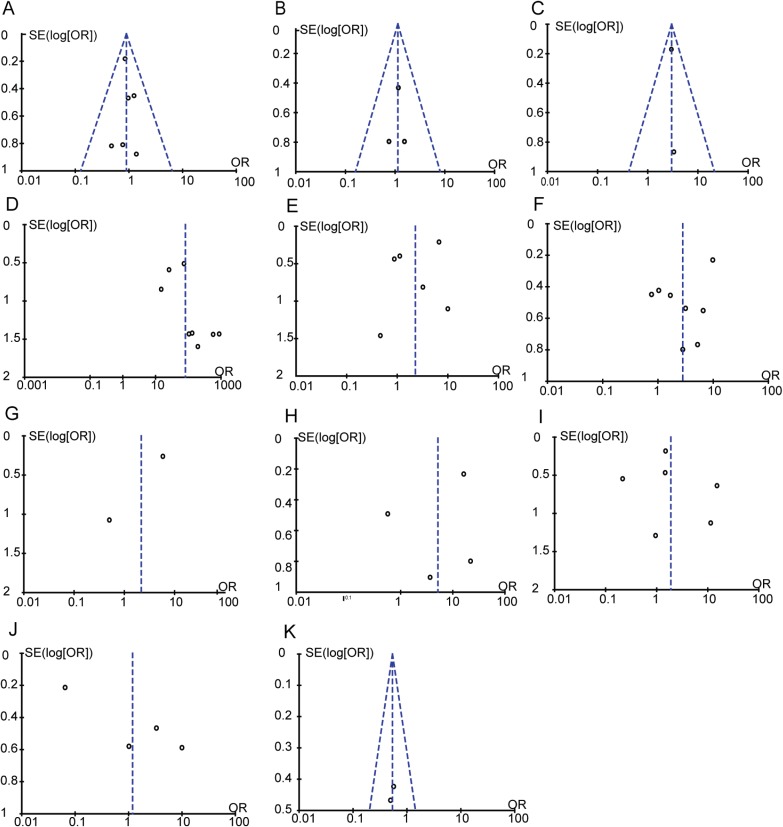
Funnel plot. (A) Overexpression of EpCAM in male group and female group. (B) Overexpression of EpCAM in age > 60 group and age ≤60 group. (C) Overexpression of EpCAM in tumour >5 cm group and tumour ≤5 cm group. (D) Overexpression of EpCAM in gastric cancer group and control group. (E) Overexpression of EpCAM in T3~T4 group and T1~T2 group. (F) Overexpression of EpCAM in LN (+) and LN (–) gastric cancer group. (G) Overexpression of EpCAM in D (+) and D (–) gastric cancer group. (H) Overexpression of EpCAM in Ⅲ~Ⅳ group and Ⅰ~Ⅱ group. (I) Overexpression of EpCAM in poorly differentiated group and well/moderate differentiated group. (J) Overexpression of EpCAM in intestinal group and diffuse group. (K) 5-year overall survival between EpCAM (+) and EpCAM (–) groups.

We detected publication bias using the RevMan 5.3 software. A funnel plot of each of the pairs of groups compared above was created with the OR as the x-axis and the SE (log[OR)]) as the y-axis. If this plot was symmetric, it would suggest that the publication bias was minimal and that the results of the present study arecredible.Begg's and Egger's tests can be used to quantify the publication biases of studies in all circumstances[23]. The results of the Begg's and Egger's tests revealed that no publication biases existed in terms of gender (Fig 13A), age (Fig 13B), tumour stage (Fig 13E), lymph node metastasis (Fig 13F), TNM stage (Fig 13H), differentiation(Fig 13I) or Lauren type (Fig 13J). Regarding tumour size (Fig 13C), distant metastasis (Fig 13G) and the 5-year overall survival rate (Fig 13K), Begg's tests revealed no publication biases, but Egger's tests could not be performed because only two studies were included. Regarding the gastric cancer group and the control group (Fig 13D), a Begg's test revealed no publication bias, but an Egger's test revealed significant publication bias.The reason for this difference in statistical significance between these two test methods might be related to the small sizes of these studies or the number of included studies and thus indicates that additional studies need to be performed (Fig 13).

## Discussion

CSCs are a subpopulation of tumour cells that have properties similar to those of normal stem cells. Stem cells are pluripotent, immune-privileged and long-living, but they depend on specialized niches. CSCs are often thought to be responsible for self-renewal, clonal tumour initiation capacity and clonal long-term repopulation potential[24]. CSCs have the ability to form tumours that recapitulate the heterogeneity of the primary tumour from which they were isolated following orthotopic transplantation into mice[25]. Both experimental models and clinical studies indicate that CSCs survive many commonly applied cancer therapeutics[26]. CSC-targeted therapies have provided us with new and promising opportunities to treat tumour cells that are resistant to current therapies and are responsible for recurrence and treatment failure[27]. Finally, the discovery of CSCs has helped us to understand the molecular mechanism of tumour genesis and development, and improvements in our understanding of the biological effects of EpCAMwill hopefully help us to develop new effective anti-EpCAM strategies.

Regarding the biological properties of CSCs, numerous studies have indicated that the assessment of the EpCAM expression level in gastric cancer tissue sections is sensible. Although many basic and clinical studies have explored the relationship between EpCAM and gastric cancer, these studies have not reached a consensus. In some reports, EpCAM overexpression is thought to be associated with improved survival, whereas in other studies, high intratumoural EpCAM expression has been identified as a poor prognostic factor[14–15,20,28]. Therefore, based on the currently available research, we performed a systematic review of the literatures with a meta-analysis to determine the associations of EpCAM expression with the genders, ages, and various clinicopathological characteristics of GC patients and to investigate the role of EpCAM in the prognoses of GC patients. The original results of meta-analyses revealed that the expression of EpCAM in the gastric cancer group was greater than that in the control group. It was found that EpCAM overexpression was related to tumour size and lymph node metastasis in GC patients. Furthermore, the GC patients who overexpressed EpCAM exhibited a lower 5-year overall survival rate than the EpCAM-negative patients. In addition, the remaining meta-analyses including gender, age, natures of the tissues, tumour stages, distant metastasis, TNM stage, differentiation and lauren type did not show significant association. The heterogeneity of the study may have a certain degree of influence on the reliability of the analysis results. Sensitivity analysis can help to find the source of heterogeneity and highlight some important limitations of empirical work. Therefore, for the significant heterogeneity studies (on tumour stages, lymph node metastases, TNM stage, differentiation and lauren type), sensitivity analysis was conducted by excluding the studies from the Chinese population or those from non-Chinese population. We had found that, for the analysis on tumour stages and lymph node metastases, the ORs of the Chinese population [6.49 (4.38, 9.63) and 6.16 (3.63, 10.44), respectively] were greater than the non-Chinese population [0.97 (0.55, 1.71) and 1.09 (0.66, 1.79), respectively]. The confidence intervals between Chinese population and non-Chinese population were completely inconsistent, and 95% CIs of the two populations were partly overlapped with their original estimate [2.26 (0.79, 6.45) and 2.78 (1.23, 6.27), respectively). For TNM stage and differentiation, the ORs were 13.69 (6.57, 28.49) and 1.58 (1.02, 2.44), respectively, and the confidence intervals overlapped with their original estimates of 5.14 (0.77, 34.37) and 1.88(0.65, 5.41). The ORs were stronger due to the removal of the heterogeneous results from Chinese populations. For the lauren type analysis, the OR was 5.39 (1.84, 15.84) and the confidence interval overlapped with the original estimate of 1.88 (0.08, 16.45). The OR was stronger due to the removal of the heterogeneous results from non-Chinese populations. In summary, sensitivity analysis had shown that the robustness of the above meta-analysis was poor, and the heterogeneity was effectively decreased after sensitivity analysis, which suggested that the heterogeneities in the original analysis were mainly due to the difference in the studies between Chinese and non-Chinese populations. It is expected that more large-scale, high-quality studies should be conducted contraposing different populations to indentify the significance of EpCAM overexpression.

In conclusion, high EpCAM expression is possibly closely related to the clinicopathological parameters of GC patients, and EpCAM might play a critical role in the pathophysiology of gastric cancer. Recent research has demonstrated that the downregulation of EpCAM significantly suppresses adhesive, invasive and migratory abilities of cell lines and tumour formation and metastatic abilities in nude mice[13,29]. Du[29] found that the proliferating cell nuclear antigen(PCNA) index of GC tissues is associated with high EpCAM expression, and EpCAM repression in GC cells can downregulatecyclin D1, the overexpression of which can lead to abnormal cellular proliferation[30]. These findings suggest that EpCAM might be associated with the proliferative activities of cancer cells and enhance the cell growth in GC that underlies the process of tumourigenesis. I SongunandMee Joo [15,17]indicated that the loss of EpCAM expression is associated with aggressive tumours, especially in GC patients with stages I and II disease, and these results are consistent with those related to ovarian carcinomas reported in the study of Kim[31]. This information may be helpful for the selection of patients who are suitable for surgery or for additional pre- or postoperative treatment. Additionally, EpCAM consistingof a 289-amino-acid-long extracellular domaincalled EpEX and a 26-amino-acidshort intracellular domain called EpICD, and Fong’s study[32] found that the loss ofmembranous EpICDexpression is a common event inhuman epithelial carcinomas. Therefore, EpCAM variants may play important roles in human malignancies, and this issue warrants further study.

In recent years, molecularly targeted therapy has provided a new strategy for clinical cancer treatment. Molecularly targeted therapy is based on the molecular level and effectively suppresses oncogenic targets. Such therapy can inhibit or kill tumour cells via its unique, steady, accurate and relentless abilities and thereby reduce the toxicity to normal cells and tissue. Successful clinical applications include the treatment of breast cancer with human epidermal growth factor receptor-2 (HER2)-specific trastuzumab and follicular non-Hodgkin’s B-cell lymphoma with CD20-specific rituximab[33]. With developments in molecular biological and immunological techniques, EpCAM-targeted cancer treatment therapy has been constantly updated. Catumaxomab, which isspecific for the EpCAM target antigen, was approved for thetreatment of malignant ascites in cancer patients with EpCAM-positive tumours in the European Union in 2009[34]. Clinical practice has indicated the remarkable efficacy of monoclonal antibodies, which have pushed cancer treatment into an unprecedented new phase. Indeed, with sufficient understanding of the biological properties of EpCAM and improvements in the technology of the production EpCAM monoclonalantibodies, EpCAM-targeted immunotherapy will likely be an exciting and promising strategy for the treatment of metastatic gastric cancer. Therefore, more prospective work needs to be conducted regarding the exact mechanisms underlying the hypothesis.

Although this meta-analysis aimed to estimate the potential correlations of the overexpression and clinical significance of EpCAM in GC patients as well as possible, it has several limitations. For example, the population of the included study were different; the numbers of studies and patients included in this meta-analysis were relatively small; the language of the study is limited to Chinese and English andthe studies employed different criteria for IHC positivity. Due to the above-mentioned limitations and the inconsistencies of the combined results, further large-scale and well-designedstudies with better exposure assessments are warranted to confirm the findings of our study and provide a higher level of evidence.

## Supporting information

S1 Fig Sensitivity analysis of tumour stages (A: Chinese population, B: non-Chinese population).
(DOCX)

S2 Fig Sensitivity analysis of lymph node metastases (A:Chinese population, B: non-Chinese population).
(DOCX)

S3 Fig Sensitivity analysis of TNM stage on Chinese population. (TIF)

S4 Fig Sensitivity analysis of differentiation on Chinese population. (TIF)

S5 Fig Sensitivity analysis of lauren type on non-Chinese population. (TIF)

S1 Table PRISMA Checklist for the Systematic Review of EpCAM. (DOCX)

S1 Text Full electronic search strategy for PUBMED database. (DOCX)
