# Supplementary figures and images for: Relationship between epithelial cell adhesion molecule (EpCAM) overexpression and gastric cancer patients: A systematic review and meta-analysis
Source: PLoS One. 2017 Apr 12;12(4):e0175357. doi: 10.1371/journal.pone.0175357 (PMC5389808; doi:10.1371/journal.pone.0175357)

**S1A:**
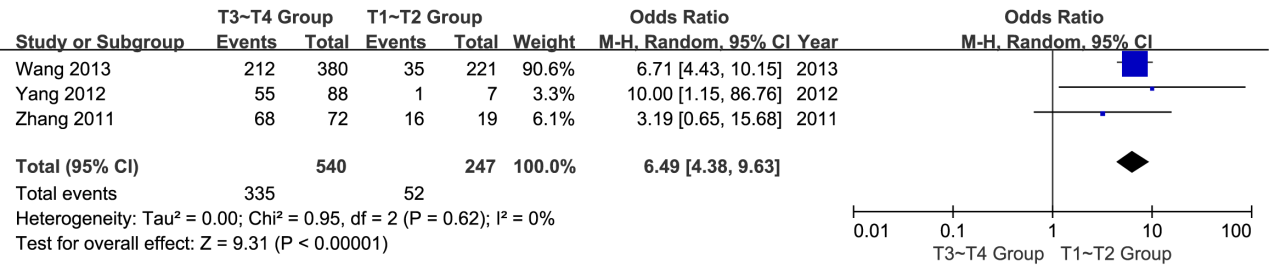


**S1B:**


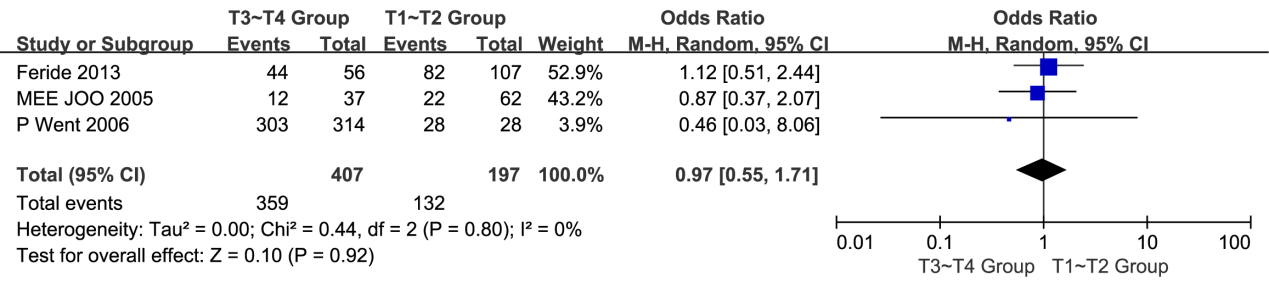

Supplement: S1 Fig — Sensitivity analysis of tumour stages (A: Chinese population, B: non-Chinese population). (DOCX) [file pone.0175357.s001.docx]

**S2A:**
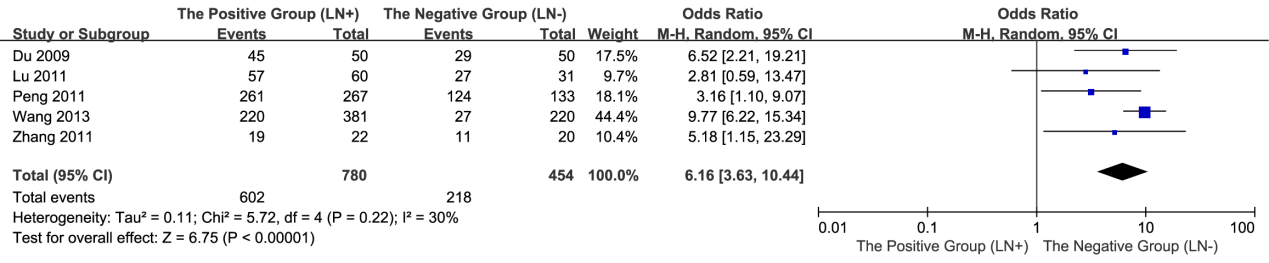


**S2B:**


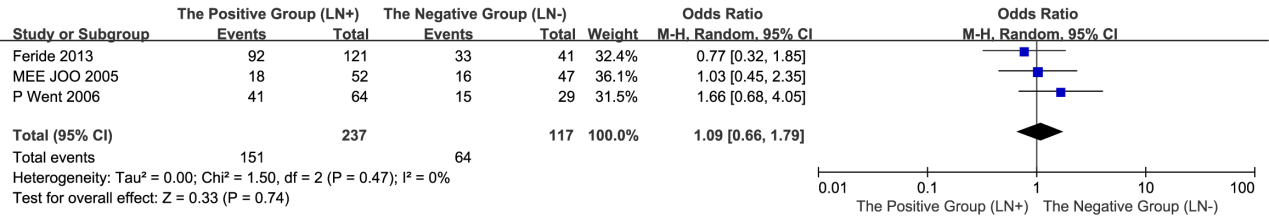

Supplement: S2 Fig — Sensitivity analysis of lymph node metastases (A:Chinese population, B: non-Chinese population). (DOCX) [file pone.0175357.s002.docx]

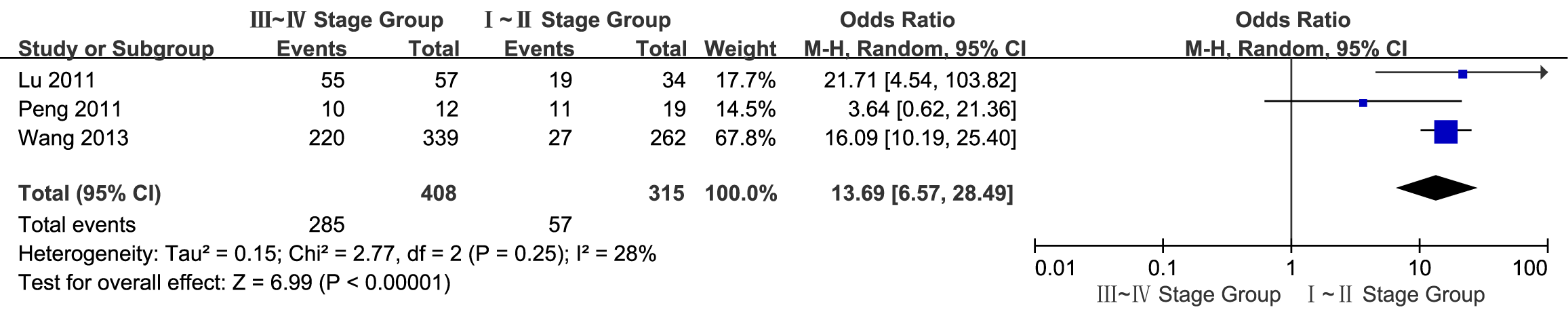

Supplement: S3 Fig — (TIF) [file pone.0175357.s003.tif]

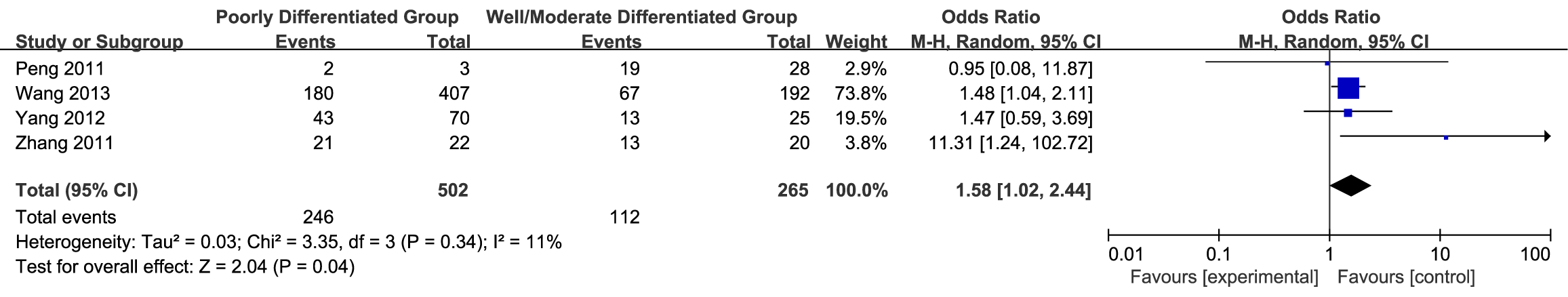

Supplement: S4 Fig — (TIF) [file pone.0175357.s004.tif]

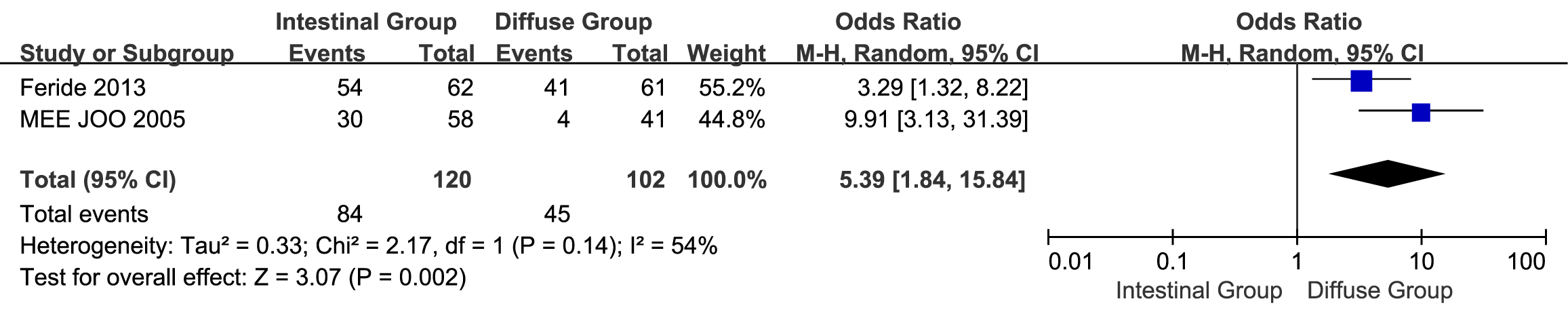

Supplement: S5 Fig — (TIF) [file pone.0175357.s005.tif]
