# Supplementary material for: Relationship between epithelial cell adhesion molecule (EpCAM) overexpression and gastric cancer patients: A systematic review and meta-analysis
Source: PLoS One. 2017 Apr 12;12(4):e0175357. doi: 10.1371/journal.pone.0175357 (PMC5389808; doi:10.1371/journal.pone.0175357)
Supplement: S1 Text — (DOCX) [file pone.0175357.s007.docx]

Related articles were identified by searching the PubMed, Cochrane Library, Medline, Web of Knowledge and CNKI databases. The detailed search strategy included the following terms in PubMed:

((EpCAM[Title/Abstract]) OR CD326[Title/Abstract]) and (((stomach[MeSH Terms]) OR gastric[Title/Abstract]) OR digestive[Title/Abstract]) and (((((neoplasm[MeSH Terms]) OR cancer[Title/Abstract]) OR tumour[Title/Abstract]) OR tumor[Title/Abstract]) OR carcinoma[Title/Abstract])
